# Opportunistic deep learning powered calcium scoring in oncologic patients with very high coronary artery calcium (≥ 1000) undergoing 18F-FDG PET/CT

**DOI:** 10.1038/s41598-022-20005-0

**Published:** 2022-11-10

**Authors:** Elisabeth Sartoretti, Antonio G. Gennari, Alexander Maurer, Thomas Sartoretti, Stephan Skawran, Moritz Schwyzer, Alexia Rossi, Andreas A. Giannopoulos, Ronny R. Buechel, Catherine Gebhard, Martin W. Huellner, Michael Messerli

**Affiliations:** 1 Department of Nuclear Medicine, University Hospital Zurich, Rämistrasse 100, 8091 Zurich, Switzerland; 2 University of Zurich, Zurich, Switzerland; 3 Institute of Diagnostic and Interventional Radiology, University Hospital Zurich, Zurich, Switzerland; 4 Health Sciences and Technology, Institute of Food, Nutrition and Health, ETH Zurich, Zurich, Switzerland; 5 Center for Molecular Cardiology, University of Zurich, Zurich, Switzerland

**Keywords:** Cardiology, Medical research, Oncology

## Abstract

Our aim was to identify and quantify high coronary artery calcium (CAC) with deep learning (DL)-powered CAC scoring (CACS) in oncological patients with known very high CAC (≥ 1000) undergoing 18F-FDG-PET/CT for re-/staging. 100 patients were enrolled: 50 patients with Agatston scores ≥ 1000 (high CACS group), 50 patients with Agatston scores < 1000 (negative control group). All patients underwent oncological 18F-FDG-PET/CT and cardiac SPECT myocardial perfusion imaging (MPI) by 99mTc-tetrofosmin within 6 months. CACS was manually performed on dedicated non-contrast ECG-gated CT scans obtained from SPECT-MPI (reference standard). Additionally, CACS was performed fully automatically with a user-independent DL-CACS tool on non-contrast, free-breathing, non-gated CT scans from 18F-FDG-PET/CT examinations. Image quality and noise of CT scans was assessed. Agatston scores obtained by manual CACS and DL tool were compared. The high CACS group had Agatston scores of 2200 ± 1620 (reference standard) and 1300 ± 1011 (DL tool, average underestimation of 38.6 ± 26%) with an intraclass correlation of 0.714 (95% CI 0.546, 0.827). Sufficient image quality significantly improved the DL tool’s capability of correctly assigning Agatston scores ≥ 1000 (*p* = 0.01). In the control group, the DL tool correctly assigned Agatston scores < 1000 in all cases. In conclusion, DL-based CACS performed on non-contrast free-breathing, non-gated CT scans from 18F-FDG-PET/CT examinations of patients with known very high (≥ 1000) CAC underestimates CAC load, but correctly assigns an Agatston scores ≥ 1000 in over 70% of cases, provided sufficient CT image quality. Subgroup analyses of the control group showed that the DL tool does not generate false-positives.

## Introduction

18F-fluorodeoxyglucose positron emission tomography/computed tomography (18F-FDG-PET/CT) is an important imaging modality for oncologic patients and provides important prognostic information, enabling improved cancer outcomes and reducing unnecessary surgery^1^. Routine 18F-FDG-PET/CT examination consists of a PET scan and a non-contrast, free-breathing, non-gated CT scan. The CT is primarily used for PET attenuation correction but also for diagnostic purposes (i.e., morphological assessment). Although the appropriate oncological diagnostic work-up and treatment is the primary concern in cancer patients undergoing 18F-FDG-PET/CT, readers should be aware of comorbidities worth reporting. In fact, most cancer patients have multiple comorbidities, including coronary heart disease (CHD)^2^.

Coronary artery calcium (CAC) is considered an important biomarker in patients with CHD. Increased CAC scores are strongly associated with cardiovascular mortality and all-cause mortality. Different CAC scores were reported to represent high-risk groups in clinical practice, but recent studies have proposed a cut-off of > 1000 as a new marker for substantial risk of cardiovascular disease outcome and mortality^3^.

While 18F-FDG-PET/CT examinations are not suited for the evaluation of CAC or coronary heart disease, an opportunistic screening resulting in the rough estimation of the coronary disease burden would be highly desirable. Specifically, the identification of the highest risk patients (CAC score > 1000) should be prioritized. Optimally, this assessment (i.e. CAC scoring) should be performed automatically and reader-independent, so that the physician can continue to focus on the oncologic workup of patients.

Recently, deep-learning (DL)-based CAC scoring tools have been developed^4–10^. These AI-backed tools enable an accurate estimation of the coronary calcium load, as measured on dedicated non-contrast ECG-gated cardiac CT scans. Most importantly, these tools alleviate the need of performing the CAC scoring manually, which results in considerable time savings for physicians^5^.

Given these considerations, we sought to test the feasibility of identifying very high CAC by means of DL powered CAC scoring in oncologic patients with known very high coronary artery calcium (≥ 1000) undergoing 18F-FDG-PET/CT. We hypothesized that an accurate estimation of the CAC load would be possible in these patients despite the fact that non-contrast, free-breathing, non-gated CT scans from 18F-FDG-PET/CT examinations were used as input data rather than dedicated non-contrast ECG-gated cardiac CT scans.

## Material and methods

This study was approved by the local ethics committee (BASEC No. 2017-01112; Kantonale Ethikkommission, Kanton Zürich, Switzerland; 28.03.2022 submission of nonsubstantial amendment, 07.04.2022 acceptance of amendment concerning the use of Coreline software) and was conducted in compliance with ICH-GCP rules and the Declaration of Helsinki. The need for informed consent was waived by the local ethics committee Kantonale Ethikkommission, Kanton Zürich, Switzerland due to the retrospective nature of the study. The study population was partially shared in a previous publication^11^.

### Study population

**Figure 1:**
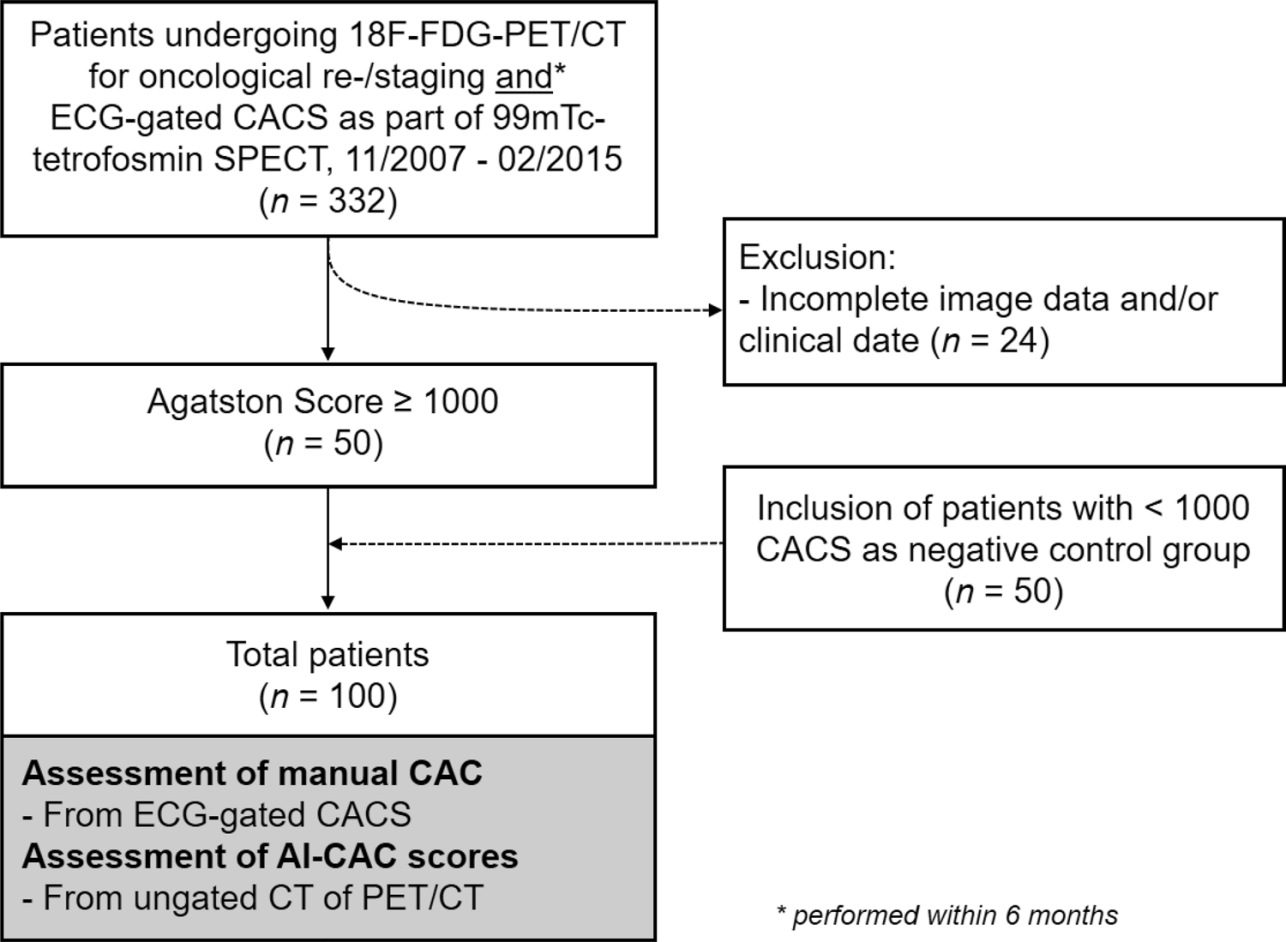
Flow chart of study. This figure describes exclusion and inclusion criteria for patients included in the study.

Our study population was selected from a retrospective cohort study of consecutive patients undergoing whole-body 18F-FDG-PET/CT for various malignant diseases at the University Hospital Zurich, Switzerland between November 2007 and February 2015. Out of 25,600 patients, 10,148 also underwent 1-day stress/rest (adenosine, dobutamine, or exercise) myocardial perfusion imaging with 99mTc-tetrofosmin single-photon emission computed tomography (SPECT-MPI), including non-contrast, ECG-gated CT for attenuation correction to evaluate known or suspected CAD. Patients who had undergone both whole-body 18F-FDG-PET/CT and SPECT-MPI within 6 months were considered eligible for inclusion into the current study. Importantly, patients were considered eligible for inclusion irrespective of type of disease, stage or therapy management. 332 patients met the inclusion criteria. From this cohort, 24 patients were excluded due to non-diagnostic image quality and/or lack of availability of clinical data. There were no further exclusion criteria. Thus, 302 patients remained. Of these, 50 with a CAC score of ≥ 1000 were identified and enrolled into the study. CAC scoring was performed manually on non-contrast ECG-gated CT scans obtained during myocardial perfusion imaging using dedicated software (Smart Score, GE Healthcare, Milwaukee, WI, USA). A flow chart of the study is presented in Fig. 1. In addition, from the same cohort of 302 patients, 50 patients with a CAC score of < 1000 (30 males, 20 females; mean age: 71 ± 7 years) were identified and enrolled into the study thereby serving as a control group to analyze whether the DL tool does not falsely overestimate the calcium score in patients, thus misclassifying them as very high-risk patients (i.e., CAC score of ≥ 1000), see Fig. 1.

### Whole-body 18F-FDG-PET/CT

Patients were instructed to fast for at least 4 h before administrating 18F-FDG. After measuring blood glucose, 18F-FDG was injected into a peripheral vein. One hour later, patients underwent PET/CT imaging from the skull to the pelvis (including a non-contrast, free-breathing, non-gated CT scan). Images were acquired in 3D mode on a Discovery VCT or Discovery RX scanner (GE Healthcare, Waukesha, WI) using well established clinical imaging protocols. The CT scan was acquired using at a tube voltage of 120 or 140 kV and with a low tube current (range 59–80 mAs). Radiation dose was 3.4 ± 0.6 mGy CT dose index volume and 422.4 ± 378 mGy*cm dose length product. Images were reconstructed using a standard soft tissue kernel and with a slice thickness and increment of 1.25 mm. PET/CT and CT images were merged and analyzed using Advantage Window volume viewer software (GE Healthcare, Milwaukee, WI, USA), as previously described^12^. The CT scan as acquired for hybrid 18F-FDG PET/CT w.

### Deep learning CAC scoring

CAC was identified and quantified using the Agatston score. Classification was performed by a fully automated DL-based CAC scoring tool (AVIEW CAC, Coreline Soft, access via https://cloud.corelinesoft.eu/login, Version v1.1.42.-win). The software was developed based on a 3-dimensional U-net architecture using non-enhanced cardiac CT scans acquired from multiple vendors and scanners. No training data were included in our study. A detailed description of this DL tool can be found elsewhere^5,8,10^.

### Image analysis

First, the non-contrast, free-breathing, non-gated CT scans from the 18F-FDG-PET/CT examination were reconstructed using the hospital’s PACS viewing system. Specifically, an image series encompassing all images from the lung apex to the lung base was generated for each patient. After anonymization of the data and images, this image series was uploaded to the cloud-based DL tool. CAC scoring was then performed automatically without further user input, and a detailed report of the results was generated.

Additionally, a board-certified radiologist (A.G.G., with 7 years of experience) graded image quality of the CT scans with respect to the heart. The following 4-point Likert scale was used: (1) poor quality, no distinction can be made between noise and calcifications, non-diagnostic; (2) insufficient quality, little distinction can be made between noise and calcifications, limited diagnostic value; (3) moderate quality, small chance of missing small calcifications, sufficient diagnostic value; (4) proper quality, unlikely that calcifications are missed, good diagnostic value. Scans rated 1 or 2 were considered non-diagnostic.

Lastly, as suggested elsewhere^13–15^, noise was measured by placing approximately 1 cm^2^ large region of interests (ROI) into the ascending aorta and into the left ventricle. From each measurement, the standard deviation (SD) was extracted, representing the noise level. The average value of these two measurements was considered representative for further analysis.

### Statistical analysis

All statistical analyses were performed with the R statistical software (version 4.0.2; R Foundation for Statistical Computing, Vienna, Austria, https://www.R-project.org/). Initially, descriptive statistics was performed on the data, and results were presented with counts and percentages. A linear regression model was fitted between the Agatston scores of the reference standard and the DL tool. Additionally, Bland–Altman analysis was performed. A two-way intraclass correlation coefficient (ICC) was computed to quantify the agreement in scores between the standard of reference and the DL tool. To quantify the impact of various variables (i.e., image quality, image noise, year of image acquisition, BMI, true Agatston score) on the accuracy of the DL tool in diagnosing an Agatston score of > 1000, a generalized linear model (GLM) was fitted. Wherever appropriate, the GLM was iteratively optimized based on the Akaike Information Criterion (AIC). Lastly, chi-square tests for independence were performed to compare the DL tool’s accuracy in diagnosing an Agatston score of > 1000, stratified by a target variable (such as image quality).

## Results

**Figure 2:**
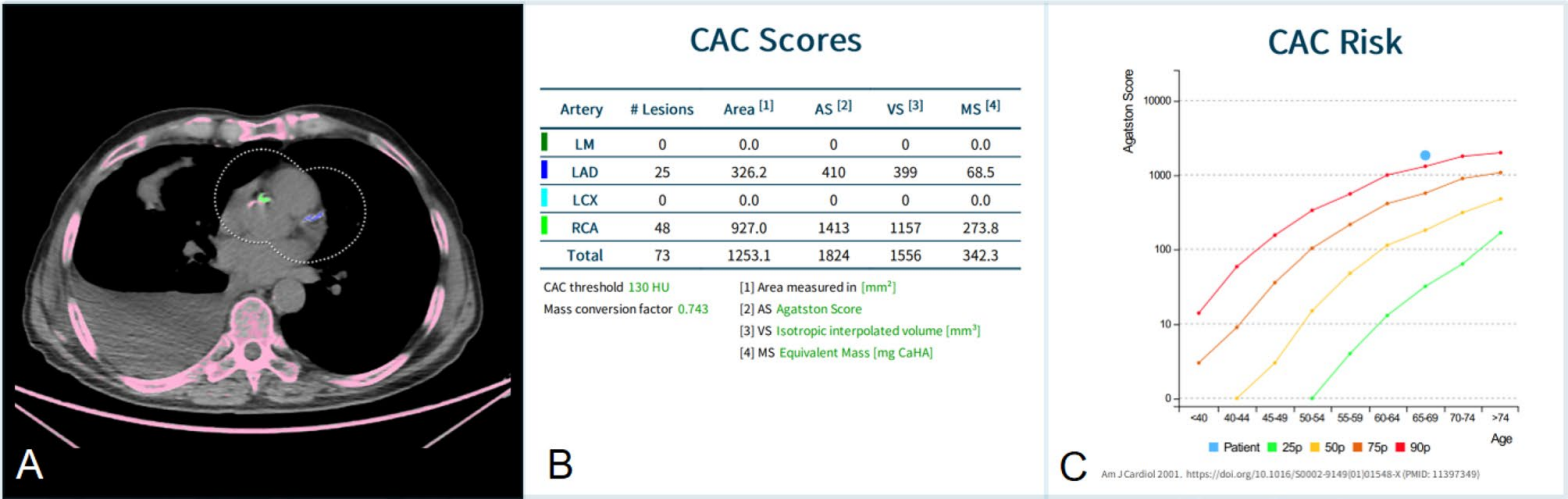
Representative CT image with automated deep learning coronary artery calcium scoring. Representative CT images of a 68-year-old man with a body mass index of 21.8 kg/m^2^ with severe coronary artery calcifications (i.e., Agatston score of 1616). Images from ungated non-contrast CT from 18F-FDG-PET/CT for staging of lung cancer (**A**), including fully automated deep learning coronary artery calcium scoring (DL-CACS) markings. Coronary calcifications in the left main (LM), left anterior descending (LAD), left circumflex artery (LCX), and right coronary artery (RCA), were correctly quantified (**B**) by the AI-CACS tool, resulting in a total score of 1824. The DL-CACS correctly attributed the highest CAC risk (**C**).

The DL tool required 87 ± 53 s to perform CACS per patient. The Agatston score was 2200 ± 1620 for the reference standard, and 1300 ± 1011 for the DL tool. Demographical information of the high CACS group (*n* = 50) is presented in Table 1. All data of the high CACS group are provided in detail in the supplementary material. A representative case of the DL tool correctly identifying the coronary calcium burden in a patient is presented in Fig. 2.

**Table 1:** Demographics of study patients (*n* = 50).

Female/male, n (%)	10 (20%)/40 (80%)
Age, years	70 ± 8.9
BMI, kg/m^2^	26 ± 5
**Oncological diagnosis, n (%)**	
Lung cancer	15 (30%)
Gastric or upper GI cancer	9 (18%)
Colorectal cancer	8 (16%)
Head and neck cancer	6 (12%)
Breast cancer	3 (6%)
Urogenital cancer	2 (4%)
Cholangiocellular carcinoma	2 (4%)
Melanoma	1 (2%)
Other	4 (8%)

Values given are mean ± standard deviation or absolute numbers and percentages in brackets.

This table describes demographics of the patients included in this study such as their oncological diagnosis, age, body mass index and gender.

*BMI* body mass index, *GI* gastrointestinal.

### Diagnostic performance of the DL tool in the high CACS group

**Figure 3:**
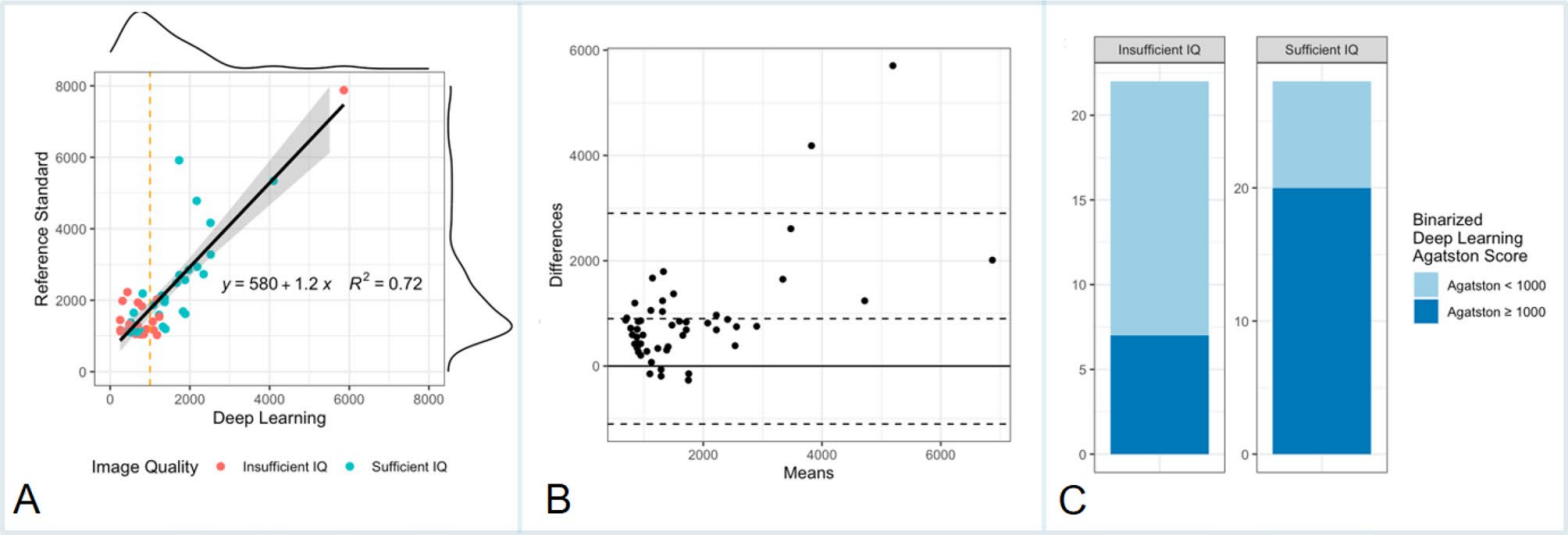
Visual representation of study data. This figure visualizes the study data, including the data of the high CACS group (n = 50): linear regression model (**A**), Bland–Altman analysis (**B**), as well as frequency of patients with a DL-based Agatston score of below or above 1000 stratified by image quality (**C**).

In the high CACS group, the Agatston score was underestimated by the DL tool in 45 cases (90%) and overestimated in 5 cases (10%) compared to the standard of reference. Specifically, the DL tool underestimated the Agatston score on average by 38.6 ± 26%. The regression model between the Agatston scores of the standard of reference and the DL tool (R^2^ = 0.72, p < 0.001) exhibited a slope of 1.2 and an intercept of 580, see Fig. 3A. Bland–Altman analysis revealed a mean difference of 900.1, a lower limit of agreement of − 1100.6 and an upper limit of agreement of 2900.8. ICC between the Agatston scores of the standard of reference and the DL tool was 0.714 (95% CI 0.546, 0.827), see Fig. 3B.

Given the importance of a CAC cut-off of 1000, we binarized the Agatston score as computed by the DL tool into < 1000 and ≥ 1000. In total, the DL tool assigned an Agatston score of < 1000 in 23 cases (i.e., false negative, 46%) and assigned an Agatston score of ≥ 1000 in 27 cases (i.e., true positive, 54%).

The accuracy of the DL tool in diagnosing an Agatston score of ≥ 1000 heavily depended on image quality (*p* = 0.02, odds ratio 4.2) and on the true (i.e., standard of reference) Agatston score (*p* = 0.02, odds ratio 1.002), but not of year of the image acquisition (*p* = 0.95), BMI of the patient (*p* = 0.61) or image noise (*p* = 0.30). Thus, a sufficient image quality and higher true Agatston scores were associated with a higher probability of the DL tool correctly assigning an Agatston score of ≥ 1000.

### Image noise and impact on DL-CACS performance

The average image noise was 15.9 ± 4.8 HU. Of all 50 patients in the high CACS group, image quality in 22 patients was deemed insufficient (i.e., image quality scores of 1 or 2), while 28 patients had sufficient image quality (i.e., image quality scores of 3 or 4). In patients with sufficient image quality, the rate of false negatives was 28.6% (i.e., 8 out of 28 patients) and the rate of true positive cases was 71.4% (i.e., 20 out of 28 patients). In contrast, in patients with insufficient image quality, the rate of false negatives was 68.2% (i.e., 15 out of 22 patients) and the rate of true positive cases was 31.8% (i.e., 7 out of 22 patients). Thus, sufficient image quality significantly (p = 0.01) and considerably improved the DL tool’s ability in correctly diagnosing an Agatston score of ≥ 1000. The data is visualized in Fig. 3C.

### Diagnostic performance of the DL-CACS tool, including negative control group

When considering all 100 patients, including the 50 patients in the control group with CAC scores of < 1000, the DL tool achieved a sensitivity of 54% (40.2%, 67.8%) and a specificity of 100% (100%, 100%) in accurately assigning patients to the two groups of CAC scores < 1000 and ≥ 1000. Importantly, the DL tool correctly assigned CAC scores of < 1000 in all 50 cases of the control group. The true Agatston score of the 50 patients in the control group was 324 ± 240 (range 0–942), while the DL tool estimated Agatston scores of 140 ± 196 (range 0–865).

## Discussion

In our retrospective study, we assessed the feasibility of quantifying coronary calcium by means of deep learning-powered CAC scoring in oncologic patients with known very high coronary artery calcium (≥ 1000) undergoing 18F-FDG-PET/CT. Our results indicate that the DL tool manages to automatically compute the Agatston score from non-contrast, free-breathing, non-gated CT datasets without any further user input. While the DL tool generally underestimated the Agatston score, the DL-tool managed to diagnose an Agatston score of ≥ 1000 in more than 70% of cases, provided the image quality of the CT scans was sufficient. Furthermore, subgroup analyses of the 50 patients in the control group showed that the DL tool seems not to generate false positive cases, since the DL tool computed Agatston scores of < 1000 in all 50 patients.

CHD is the most common cause of death worldwide. An early and accurate risk stratification in patients at risk of CHD is highly recommended. CACS is a proven reliable method for CHD risk assessment and is thus featured in several guidelines, including the recent 2021 guideline of the European Heart Association^9,16^. In patients with additional oncologic disease burden, potential CHD burden should be addressed early and aggressively to avoid further risk of additional comorbidity or even mortality from cardiovascular disease events.

The Agatston score as measured during CAC scoring provides an estimation of the coronary atherosclerotic burden. In clinical practice, patients with an Agatston score of > 300 or > 400 are classified as high-risk individuals. A recent analysis from the MESA (Multi-Ethnic Study of Atherosclerosis; a study on primary prevention patients) trial indicates, however, that individuals with a CAC score of > 1000 constitute a unique population with a substantially higher risk of cardiovascular disease (CVD) events, non-cardiovascular disease outcomes, and mortality compared to those with lower CAC score. Specifically, these patients exhibited an annualized 3-point major adverse cardiovascular event rate of 3.4 per 100 person years, which is comparable to the rate reported for stable treated secondary prevention patients. Furthermore, these patients are at an almost 2 times increased risk for all CVD and all CHD events, and an almost 1.5 times increased risk for non-CVD events compared with those with a CAC score of 400–999. In these very high CAC score patients, an aggressive prevention strategy with pharmacological agents, such as statins, ezetimibe, and proprotein convertase subtilisin/kexin type 9 inhibitors should be implemented immediately upon diagnosis^3^.

An opportunistic screening aiming at identifying patients with a CAC score of > 1000 is highly desirable, especially in patients with multiple comorbidities, such as cancer patients. For the latter, the management of CAD is of special clinical interest as patients undergoing cytotoxic chemotherapy may have further risk of CVD events. To the best of our knowledge, this is the first study assessing the use of deep learning based CACS using the CT component of 18F-FDG-PET/CT in patients with known very high CAC score of > 1000.

Currently, calcium scoring is performed by manually delineating calcifications using dedicated software. CAC quantification accuracy is then largely dependent on the accuracy of the manual measurements and the type of CT scan. For accurate CAC estimation, a non-contrast prospective ECG-triggered cardiac CT scan performed in breath-hold is recommended. This ensures that the influence of cardiac motion and respiratory artifacts, potentially obscuring small anatomical details and calcifications, can be minimized^6,9^.

In theory, however, CAC can be scored on any non-contrast CT, but at the risk of underestimating the calcium load^9,17–20^. When the CT scan is performed in free-breathing and without ECG-triggering minor calcifications will not be visible. Furthermore, the underestimation of the calcium load increases in patients with high (i.e., > 400) CAC^18^. In our study, we only included patients with known very high CAC score (> 1000) for the study group, and observed a general underestimation of CAC. Based on the considerations outlined above, we attribute this underestimation mainly to the usage of non-gated CT scans and to the selected patient group with very high CAC, rather representing a general shortcoming of our DL tool. Importantly, we also observed that image quality of the CT scan had a large impact on the accuracy of our DL tool in identifying patients with an Agatston score of > 1000. In cases where image quality is further impaired by heavy artifacts, an accurate estimation of the CAC load becomes even more difficult.

Additionally, it should be noted that CAC scoring is a repetitive and time-consuming task. Artificial intelligence-based solutions enabling an automated estimation of CAC have been developed^4–10^. Promising results have been published, showing that CAC can be estimated accurately with AI-based solutions relative to manual measurements. For example, Vonder et al. reported an ICC of 0.958 between AI-based and manual measurements in 997 patients undergoing low-dose ECG-gated cardiac CT for calcium scoring^5^. While the ICC was lower in this study (0.741), potentially due to the aforementioned reasons, we can confirm the convenience of DL-powered CACS. The tool can be programmed in such a manner that the CT scan is automatically uploaded to the cloud-based interface. Then, without further user input, the tool performs CACS within 87 ± 53 s. and generates a detailed report of the results. The physician can then focus on the actual interpretation of the PET/CT scan, without the need for a potentially time-consuming analysis that is not directly related to the oncologic work-up in the foreground.

Lastly, we would like to point out that we used the DL tool directly, without any additional training on our data or other data beforehand. Our data rather represents a true external validation set. Moreover, we used images acquired several years ago (back to 2007), and the DL tool was still able to achieve comparably good results, especially when the image quality was sufficient.

Our study has the following limitations: first, this was a retrospective, single-center study with a limited number of subjects. Notably, our patient cohort was also quite heterogeneous with patients being included irrespective of their type of disease, disease stage or their therapy management. The heterogeneity of our study cohort may have impacted our results, as the risk of CAD may be distributed very unevenly between individuals included in our study. Second, PET/CT and cardiac SPECT/CT were performed within a time interval of 6 months. This may have led to changes in the CAC burden. Although such changes might be rather minor, it may have impacted our results. Third, we did not assess the influence of the scanning parameters on the performance of the DL tool.

In conclusion, our study indicates that a DL tool can automatically quantify coronary calcium in oncologic patients with known very high coronary artery calcium (≥ 1000) undergoing 18F-FDG-PET/CT. The DL tool manages to automatically compute the Agatston score from free-breathing non-contrast, non-gated CT datasets used for PET attenuation correction without any further user input. Importantly, in patients with known very high CAC (≥ 1000), the DL tool enables the diagnoses of an Agatston score of ≥ 1000 in more than 70% of cases, provided sufficient CT image quality. Furthermore, subgroup analyses of the control group showed that the DL tool does not generate false positive cases, thus leading to 100% specificity of the tool in classifying patients into CAC of < 1000 and ≥ 1000.

## Supplementary Information

Supplementary Information.

## Data Availability

Data can be made available upon reasonable request to the corresponding author.

## References

[1] Hess S, Blomberg BA, Zhu HJ (2014). The PIVOTAL role of FDG-PET/CT in modern medicine. Acad. Radiol..

[2] Sturgeon KM, Deng L, Bluethmann SM (2019). A population-based study of cardiovascular disease mortality risk in US cancer patients. Eur. Heart J..

[3] Peng AW, Dardari ZA, Blumenthal RS (2021). Very high coronary artery calcium (≥1000) and association with cardiovascular disease events, non-cardiovascular disease outcomes, and mortality: Results from MESA. Circulation.

[4] Winkel DJ, Suryanarayana VR, Ali AM (2021). Deep learning for vessel-specific coronary artery calcium scoring: Validation on a multi-centre dataset. Eur. Heart J. Cardiovasc. Imaging.

[5] Vonder M, Zheng S, Dorrius MD (2021). Deep learning for automatic calcium scoring in population-based cardiovascular screening. JACC Cardiovasc. Imaging.

[6] Eng D, Chute C, Khandwala N (2021). Automated coronary calcium scoring using deep learning with multicenter external validation. NPJ Digit. Med..

[7] van Velzen SGM, Lessmann N, Velthuis BK (2020). Deep learning for automatic calcium scoring in CT: Validation using multiple cardiac CT and chest CT protocols. Radiology.

[8] Lee J-G, Kim H, Kang H (2021). Fully automatic coronary calcium score software empowered by artificial intelligence technology: Validation study using three CT cohorts. Korean J. Radiol..

[9] Xu J, Liu J, Guo N (2021). Performance of artificial intelligence-based coronary artery calcium scoring in non-gated chest CT. Eur. J. Radiol..

[10] Sartoretti T, Gennari AG, Sartoretti E (2022). Fully automated deep learning powered calcium scoring in patients undergoing myocardial perfusion imaging. J. Nucl. Cardiol..

[11] Haider A, Bengs S, Schade K (2020). Myocardial 18F-FDG uptake pattern for cardiovascular risk stratification in patients undergoing oncologic PET/CT. JCM.

[12] Messerli M, Stolzmann P, Egger-Sigg M (2018). Impact of a Bayesian penalized likelihood reconstruction algorithm on image quality in novel digital PET/CT: Clinical implications for the assessment of lung tumors. EJNMMI Phys..

[13] Gebhard C, Fiechter M, Fuchs TA (2013). Coronary artery calcium scoring: Influence of adaptive statistical iterative reconstruction using 64-MDCT. Int. J. Cardiol..

[14] Tesche C, De Cecco CN, Schoepf UJ (2017). Iterative beam-hardening correction with advanced modeled iterative reconstruction in low voltage CT coronary calcium scoring with tin filtration: Impact on coronary artery calcium quantification and image quality. J. Cardiovasc. Comput. Tomogr..

[15] van Osch JAC, Mouden M, van Dalen JA (2014). Influence of iterative image reconstruction on CT-based calcium score measurements. Int. J. Cardiovasc. Imaging.

[16] Visseren FLJ, Mach F, Smulders YM (2022). 2021 ESC Guidelines on cardiovascular disease prevention in clinical practice. Eur. J. Prev. Cardiol..

[17] Takx RAP, de Jong PA, Leiner T (2014). Automated coronary artery calcification scoring in non-gated chest CT: Agreement and reliability. PLoS One.

[18] Mylonas I, Kazmi M, Fuller L (2012). Measuring coronary artery calcification using positron emission tomography-computed tomography attenuation correction images. Eur. Heart J. Cardiovasc. Imaging.

[19] Fan R, Shi X, Qian Y (2018). Optimized categorization algorithm of coronary artery calcification score on non-gated chest low-dose CT screening using iterative model reconstruction technique. Clin. Imaging.

[20] Xia C, Vonder M, Pelgrim GJ (2021). High-pitch dual-source CT for coronary artery calcium scoring: A head-to-head comparison of non-triggered chest versus triggered cardiac acquisition. J. Cardiovasc. Comput. Tomogr..

